# Enhancing Immune Responses to a DNA Vaccine Encoding *Toxoplasma gondii* GRA7 Using Calcium Phosphate Nanoparticles as an Adjuvant

**DOI:** 10.3389/fcimb.2021.787635

**Published:** 2021-12-16

**Authors:** Hong-Chao Sun, Jing Huang, Yuan Fu, Li-Li Hao, Xin Liu, Tuan-Yuan Shi

**Affiliations:** ^1^ Department of Animal Parasitology, Institute of Animal Husbandry and Veterinary Medicine, Zhejiang Academy of Agricultural Science, Hangzhou, China; ^2^ Department of Animal Epidemic Surveillance, Zhejiang Provincial Animal Disease Prevention and Control Center, Hangzhou, China; ^3^ College of Life Science and Technology, Southwest Minzu University, Chengdu, China

**Keywords:** *Toxoplasma gondii*, DNA vaccine, dense granule protein 7 (GRA7), calcium phosphate nanoparticles (CaPNs), immune response

## Abstract

*Toxoplasma gondii* infects almost all warm-blooded animals, including humans. DNA vaccines are an effective strategy against *T. gondii* infection, but these vaccines have often been poorly immunogenic due to the poor distribution of plasmids or degradation by lysosomes. It is necessary to evaluate the antigen delivery system for optimal vaccination strategy. Nanoparticles (NPs) have been shown to modulate and enhance the cellular humoral immune response. Here, we studied the immunological properties of calcium phosphate nanoparticles (CaPNs) as nanoadjuvants to enhance the protective effect of *T. gondii* dense granule protein (GRA7). BALB/c mice were injected three times and then challenged with *T. gondii* RH strain tachyzoites. Mice vaccinated with GRA7-pEGFP-C2+nano-adjuvant (CaPNs) showed a strong cellular immune response, as monitored by elevated levels of anti-*T. gondii*-specific immunoglobulin G (IgG), a higher IgG2a-to-IgG1 ratio, elevated interleukin (IL)-12 and interferon (IFN)-γ production, and low IL-4 levels. We found that a significantly higher level of splenocyte proliferation was induced by GRA7-pEGFP-C2+nano-adjuvant (CaPNs) immunization, and a significantly prolonged survival time and decreased parasite burden were observed in vaccine-immunized mice. These data indicated that CaPN-based immunization with *T. gondii* GRA7 is a promising approach to improve vaccination.

## Introduction

*Toxoplasma gondii*, the causative agent of toxoplasmosis (Kato, 2018), is an Apicomplexa phylum parasite with a broad host range and worldwide distribution. *T. gondii* can infect almost all homeothermic animals including humans (Prandovszky et al., 2018; Coutermarsh-Ott, 2019). Although most infections are asymptomatic, the pathogen can cause severe disease manifestations and even death in immunocompromised individuals and significant economic losses to the livestock industry (Wang et al., 2017). *T. gondii* infection is acquired by consumption of raw or undercooked meat containing tissue cysts and food or water contaminated with oocysts shed from cats (Mévélec et al., 2020). Currently, there are no effective vaccines against toxoplasmosis, and treatment relies on the use of drug therapies. However, all treatments affect only tachyzoites and are ineffective against *T. gondii* cysts in tissues. Furthermore, antiparasitic drugs cause serious adverse side effects and produce drug-resistant parasite strains (Dunay et al., 2018). Therefore, a safe and effective vaccine formulation that prevents *T. gondii* infection is needed. Many antigens have been identified as vaccine candidates in the last few years (Zhang et al., 2013; Zhang et al., 2015; Montazeri et al., 2017; Wang et al., 2019). The cellular immune response plays a major role in controlling both acute and chronic *T. gondii* infection. Interleukin (IL)-12 is generated by innate immune cells to protect against *T. gondii* infection and is essential for the regulation of interferon gamma (IFN-γ) (Aliberti, 2005). Among the vaccine candidates, dense granule protein (GRA7) induces a strong antibody response during acute infection (Quan et al., 2012) and strong humoral and cellular immunity responses against *T. gondii* infection (Verhelst et al., 2011; Selseleh et al., 2012); therefore, GRA7 is an attractive vaccine candidate against *T. gondii*.

In recent years, DNA vaccines, such as GRA4 (Zhang et al., 2007), ROP29 (Lu et al., 2018), and GRA2 (Ching et al., 2016), have been of great interest in immunization against *T. gondii* infection. Although DNA vaccines produced a better immune response, these vaccines have often been poorly immunogenic, and it is critical to optimize the pathways of delivery for an optimal vaccination strategy (Min et al., 2012). Nanoparticles (NPs) as vaccine adjuvants have been shown to enhance humoral and immune responses, and the use of novel NP technologies can induce CD8^+^ T-cell immunity responses (Wilson et al., 2015). Calcium phosphate nanoparticles (CaPNs) and aluminum hydroxide (alum) have been used as vaccine adjuvants (effective antigen delivery systems) for many years and have several advantages, such as biocompatibility, safety, effective delivery of antigens to specific locations, and robust humoral and cellular responses (Lin et al., 2017).

In this study, a DNA vaccine using *T. gondii* GRA7 was designed and encapsulated in CaPNs, which has never been previously evaluated to our knowledge. The objective of this study was to assess the immunogenic and protective efficacy of the GRA7-pEGFP-C2+nano-adjuvant (CaPNs) vaccine.

## Materials and Methods

### Mice and Parasites

BALB/c mice aged between 6 and 8 weeks were purchased from the Laboratory Animal Centre of Zhejiang Academy of Agricultural Sciences. All the mice were maintained under specific pathogen-free standard conditions with stable temperature (24°C ± 1°C), 50% ± 10% humidity, and a 12/12-h light–dark cycle; food and water were supplied *ad libitum*. All experiments were approved by the Animal Ethics Committee of Zhejiang Academy of Agricultural Sciences. BALB/c mice were used for the vaccination study, and Vero cells were used for maintenance and proliferation of *T. gondii* RH strain tachyzoites.

### Preparation of *Toxoplasma gondii* Antigen (*Toxoplasma* lysate antigen)

*Toxoplasma* lysate antigen (TLA) was obtained as previously described (Holec-Gasior et al., 2010). Briefly, 10^7^ tachyzoites were collected from Vero cells, washed three times with sterile phosphate buffered saline (PBS), and then centrifuged at 1,000 rpm for 10 min. The tachyzoites were disrupted using 10 freezing cycles at -80°C and thawing at 37°C. Then, the supernatant with TLA was collected, its concentration was measured using a bicinchoninic acid (BCA) Protein Assay Kit (Sangon Biotech, Shanghai, China), and it was stored at -80°C until use.

### Plasmid Preparation

A total of 10^7^ *T. gondii* tachyzoites were collected, and total RNA was extracted using TRIzol reagent according to the manufacturer’s instructions and then reverse transcribed into cDNA using the First Strand cDNA Synthesis Kit. The whole Coding sequence (CDS) of GRA7 was amplified from cDNA using PCR with primers containing *EcoRI* and *BamHI* restriction sites (underlined), 5′-gaattcATGGCCCGACACGCAATT-3′ (forward) and 5′-ggatccCTGGCGGGCATCCTCCCCATCTT-3′ (reverse). PCR amplification was performed as follows: 95°C for 5 min, followed by 35 cycles of 95°C for 30 s, 55°C for 30 s, and 72°C for 1 min, with a final extension time of 72°C for 10 min. The PCR product was detected by 1.5% agarose gel electrophoresis, the target band was purified and cloned into the pMD-19T vector, and the clone was sequenced by Sangon Biotech Company (Shanghai). The correct GRA7-pMD-19T sequence was cloned into the eukaryotic expression plasmid pEGFP-C2 using *EcoRI* and *BamHI* restriction enzymes. The recombinant plasmid GRA7-pEGFP-C2 was extracted using a Plasmid Purification Kit (Solarbio, China, Beijing), and its concentration was measured using a NanoDrop2000 Ultra Micro Spectrophotometer. Then, the preparation plasmid was stored at -20°C until use.

### Recombinant Plasmid Expression in Vero Cells

Vero cells were cultured in Dulbecco’s modified Eagle’s medium (DMEM) with 100 μg/ml streptomycin/penicillin and 10% fetal bovine serum (FBS) at 37°C and 5% CO_2_. Vero cells were cultivated in six-well plates with cell slides before transfection, and then the recombinant plasmid GRA7-pEGFP-C2 (4 μg) or the empty plasmid (pEGFP-C2) was transfected into Vero cells using 10 μl LipoFiterTM Liposomal Transfection Reagent (Hanbio Biotechnology, Shanghai, China). After inoculation for 48 h, the cell climbing tablets were removed from the six-well plates and washed with 0.1 M PBS three times and then fixed in 4% paraformaldehyde for 15–20 min. Fifty microliters of 2-(4-amidinophenyl)-6-indolecarbamidine dihydrochloride (DAPI; Beyotime, Shanghai, China) was added to the climbing tablets and incubated for 5 min. Finally, the expression of GRA7 in Vero cells was observed using a laser confocal microscope.

GRA7 protein expression from the Vero cells was analyzed by Western blot as follows. Vero cells transfected with GRA7-pEGFP-C2 and pEGFP-C2 were collected, and protein was isolated with radioimmunoprecipitation assay (RIPA) lysis buffer containing 1 mM phenyl-methanesulfonyl fluoride (PMSF; Beyotime Biotechnology, China), then the lysis solution was centrifuged at 12,000 rpm for 10 min at 4°C. Next, the protein was separated by sodium dodecyl sulfate-polyacrylamide gel electrophoresis (SDS-PAGE) and transferred onto a polyvinylidene fluoride (PVDF) membrane by electric transfer instrument (Bio-Rad, America). The membrane was sealed overnight at 4°C with 5% skimmed milk after washing three times with PBS with 0.05% Tween-20 (PBST), then coated with anti-*T. gondii* tachyzoite antigen mouse sera (diluted 1:1,000), followed by incubation for 2 h with a horseradish peroxidase (HRP)-labeled goat anti-mouse IgG antibody (Solarbio, China). Finally, the bands were detected using enhanced chemiluminescence (ECL; Thermo, America).

### Nanoparticle Synthesis and Calcium Phosphate Nanoparticle-Coated DNA Vaccine

CaPNs were prepared as previously described (He et al., 2000). Briefly, 12.5 mM dibasic sodium phosphate, 12.5 mM calcium chloride, and 15.6 mM sodium citrate were mixed together slowly and stirred for 48 h. After sonication for 30 min, a dynamic light-scattering instrument (Anton Paar Litesizer 500) and transmission electron microscope were used to determine the average size distribution, and the particle morphology was observed by scanning electron microscopy (SEM). Subsequently, a GRA7 DNA vaccine coated with CaPNs was prepared by vortexing mixtures of GRA7-pEGFP-C2 and CaPNs for 60 min, with 100 μg plasmid plus 100 μg NPs. The particles were isolated by ultracentrifugation at 66,000 g for 30 min, the supernatant was collected, the particles were redispersed in 1 ml sterile ultrapure water, and the uncoated plasmid was removed by this purification method. Afterward, the concentration of the GRA7-pEGFP-C2 plasmid in the supernatant or in the particles was analyzed using a NanoDrop2000 Ultra Micro Spectrophotometer. The loading efficiency (LE) was determined using the following equation:


$$\begin{array}{c} \text{LE}\%=(\text{Total amount of plasmid–free plasmid})/\text{Total amount of plasmid}\;\\ \times \;100.\;\text{A sample with non–loaded CaPNs was applied as a negative blank}. \end{array}$$

### Immunization and Challenge

A total of five groups of female BALB/c mice (13 mice/group) were used for the immunization experiment, and these mice were injected three times with 100 μl of the purified GRA7-pEGFP-C2 plasmid DNA dissolved in 100 μl sterile 0.1 M PBS, empty vector (pEGFP-C2), or GRA7-pEGFP-C2+nano-adjuvant (CaPNs). At the same time, two control groups (PBS and CaPNs) were designed. For the second and third inoculations, mice were boosted using the same protocol on days 14 and 28. Tail blood was collected from each mouse on days 0, 14, 28, 42, and 63, and sera were obtained and stored at -20°C until use.

Two weeks after the last immunization, 10 mice from each group were intraperitoneally injected with *T. gondii* RH strain tachyzoites (1 × 10^4^/each) as previously described (Han et al., 2017; Song et al., 2020). The status of infected mice was monitored every day, and the survival rate was recorded.

### Determination of Immunoglobulin G Titer and Subclasses

To investigate the humoral immune response induced in all immunized mice, total immunoglobulin G (IgG), IgG1, and IgG2 were measured using enzyme-linked immunosorbent assays (ELISAs) according to the manufacturer’s instructions (MultiSciences, Hangzhou, China). Briefly, 96-well microplates were coated with 100 μl/well of TLA (20 μg/ml) (Ahmadpour et al., 2017; Roozbehani et al., 2018). The microplates were blocked with 100 μl 5% skimmed milk in PBST for 2 h after overnight coating. Then, 100 μl of mouse serum (diluted 1:100 in 1% skimmed milk) was added to each well and incubated for 1 h at 37°C. After washing three times with PBST, the wells were incubated with HRP-conjugated anti-mouse IgG (diluted 1:2,000 in 1% skimmed milk), IgG1 (1:2,000), and IgG2a (1:2,000) for 40 min at 37°C. After five times of washing, a Tetramethylbenzidine (TMB) substrate solution was added and incubated for 15 min at 37°C and then was stopped by the addition of 2 M H_2_SO_4_. Finally, optical density (OD) values were measured at 450 nm. All samples were run in triplicate.

### Lymphocyte Proliferation Assay and Cytokine Assay

Two weeks after the last immunization, three mice from each group were euthanized, and splenocytes were collected and treated with red blood cell lysate and then cultured in a 96-well plate (1 × 10^5^ cells/well) in DMEM (100 μg/ml streptomycin/penicillin and 10% FBS). Thereafter, the cells were stimulated with 10 μg/ml TLA or 7.5 μg/ml concanavalin A (ConA) (positive control). As a negative control, media alone were added. The plates were incubated at 37°C in 5% CO_2_ for 72 h, after which Cell Counting Kit (CCK)-8 solution was added (50 μl/well) and cultured for 4 h. Proliferative activity was evaluated by measuring the OD values at 450 nm using an ELISA reader. The splenocyte stimulation index (SI) was calculated as the ratio of the average absorbance of the TLA-treated samples to the average absorbance of the negative groups. All samples were run in triplicate.

Splenocytes were collected as mentioned above and cultured in 96-well microtiter plates. The supernatants were harvested and assayed for IL-4 at 24 h, IL-10 at 72 h, and IL-12 and interferon gamma (IFN-γ) at 96 h using ELISA kits according to the manufacturer’s instructions.

### Determination of Parasite Burden

In order to evaluate tissue parasite burden, the heart, liver, spleen, and lung from three mice (each group) were removed. We collected the tissues using sterile scissors, and the tissues were divided into masses of equal quality (1 mg). Genomic DNA was extracted using the genomic DNA extraction kit (TIANGEN, Beijing, China) according to the manufacturer’s instructions. Afterward, the parasite burdens were determined by quantitative real-time PCR using the repeated element (RE) primers (forward, 5-AGGGACAGAAGTCGAAGGGG-3; reverse, 5- GCAGCCAAGCCGGAAACATC-3) (Roozbehani et al., 2018). The final volume of the Q-PCR reaction was 20 μl containing 10 μl SYBR green master mix (TAKARA, Japan), 0.5 μl forward primer (10 pmol), 0.5 μl reverse primer (10 pmol), 1 μl DNA template, 8 μl RNase-free water. The amplification steps were an initial denaturation at 95°C for 10 min, and amplification consisted of 40 cycles of denaturation at 95°C for 15 s, annealing at 60°C for 30 s, and amplification at 72°C for 30 s. Melting curve analysis was performed to verify the specific amplification of the correct sequence. The standard curve was determined by the known concentration of the *T. gondii* RH tachyzoites DNA. The number of parasites in the samples was calculated from the threshold cycle (Ct) value according to the standard curve (Y = -3.48X + 32.326; R^2^ = 0.987). The results were based on three independent experiments.

### Statistical Analysis

All statistical analyses were performed using GraphPad Prism Version 5. Antibody production and cytokine levels were analyzed using one-way ANOVA. Tukey’s Student range test was used when a significant difference appeared. *P* value of <0.05 was considered a significant difference.

## Results

### Expression of the Recombinant Plasmid

**Figure 1:**
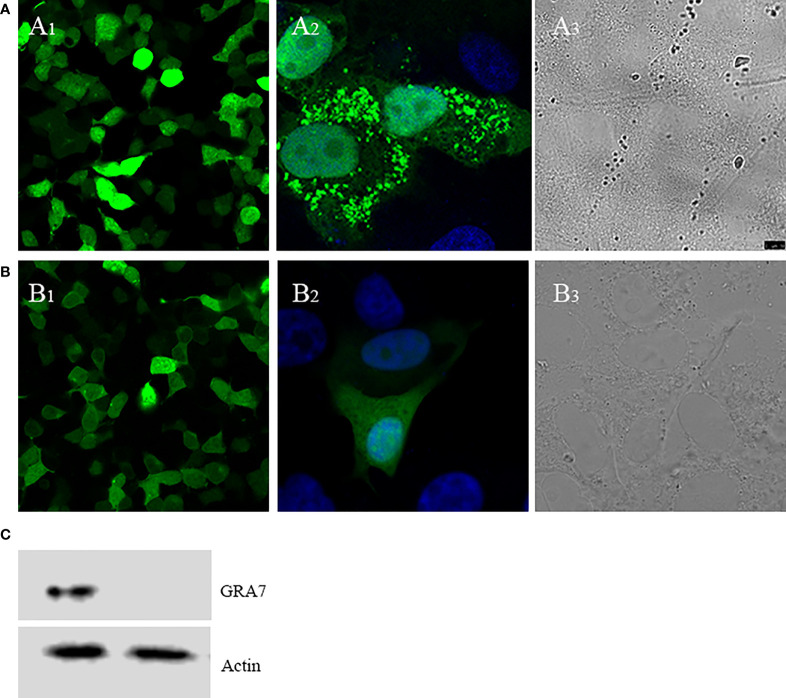
Direct fluorescence detection of the GRA7-EGFP-C2 fusion protein in transfected Vero cells. **(A)** Cells transfected with GRA7-pEGFP-C2 were detected under blue light **(A1)**; the localization of GRA7 in Vero cells was observed under blue light **(A2)** and white light **(A3)**. **(B)** Cells transfected with pEGFP-C2 were detected under blue light **(B1)**; single cells transfected with pEGFP-C2 were observed under blue light **(B2)** and white light **(B3)**. **(C)** Western blot analysis of GRA7 protein recognized by anti-*Toxoplasma gondii* mouse sera.

The expression and localization of GRA7 in Vero cells and cells transfected with pEGFP-C2 were analyzed using laser confocal microscopy (**Figures 1A, B**). Green fluorescence was observed in the GRA7-pEGFP-C2 (**Figures 1A1, A2**)- and pEGFP-C2-transfected groups (**Figures 1B1, B2**). A2 and B2 showed the single cell transfected with GRA7-pEGFP-C2 and pEGFP-C2 and A3 and B3 exhibited the cells transfected with GRA7-pEGFP-C2 pEGFP-C2 (**Figures 1A3, B3**), which were detected under white light, whereas no fluorescence was observed in the untransfected cells. GRA7 protein expression in the transfected Vero cells was determined by Western blot analysis; as shown in **Figure 1C**, a specific band was detected in lysates of the GRA7-pEGFP-C2-transfected cells, whereas the negative control cells showed no bands. These results indicated that the GRA7-pEGFP-C2 recombinant plasmid was successfully transfected and expressed in Vero cells.

### Synthesis of Calcium Phosphate Nanoparticles and Preparation of Nanoparticle-Coated DNA Vaccines

**Figure 2:**
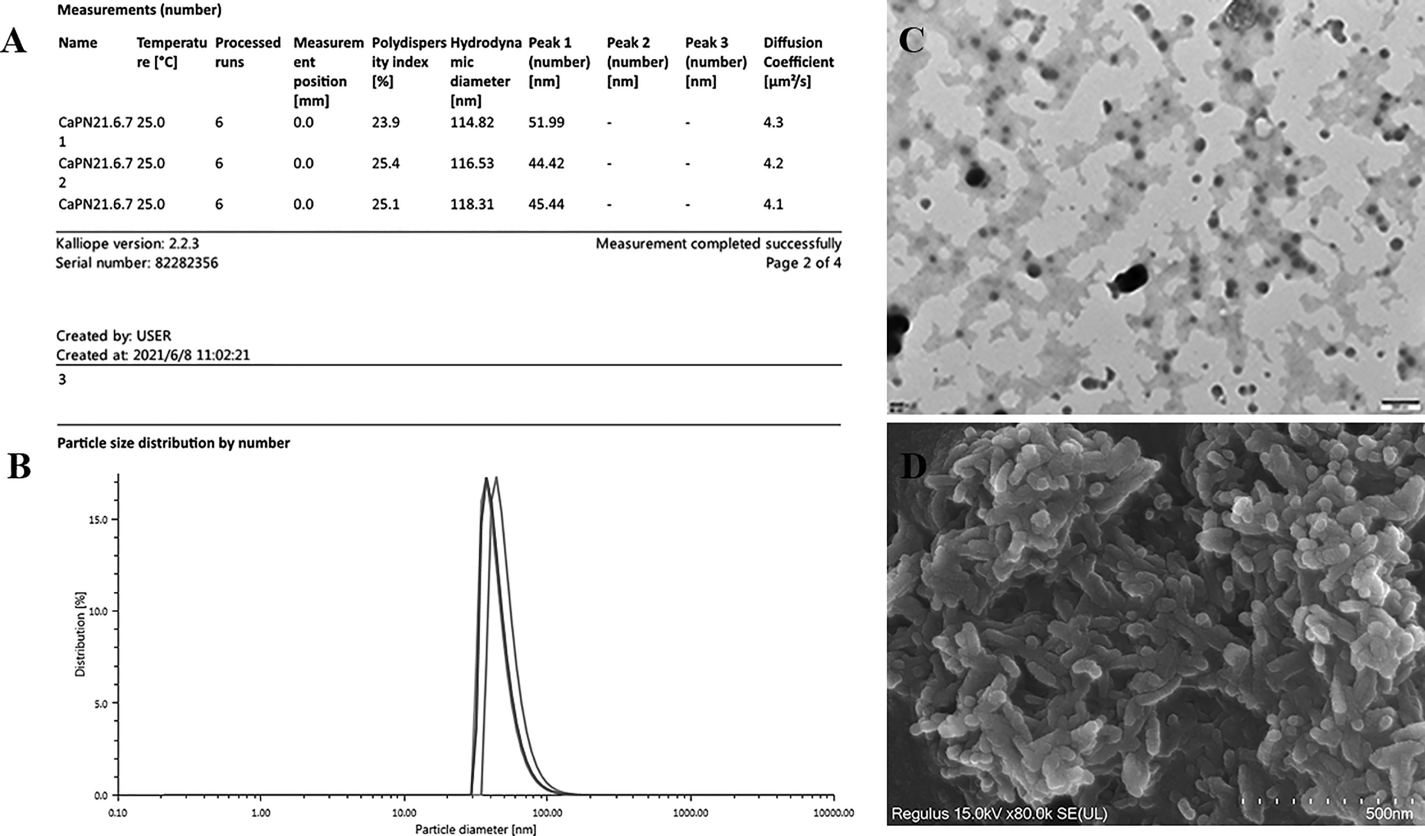
Characterization of calcium phosphate nanoparticles (CaPNs). **(A)** Morphology was observed using scanning electron microscopy (SEM). **(B)** Nanoparticle size and distribution were observed using transmission electron microscopy (TEM). **(C)** Nanoparticle size and diffusion coefficient were analyzed using an Anton Paar Litesizer 500. **(D)** Particle size distribution by number is shown.

The average NP diameter was 47.28 nm, and the diffusion coefficient was approximately 4, indicating acceptable monodispersity (**Figures 2A, B**); a consistent size was observed by transmission electron microscopy (TEM) (**Figure 2C**). The morphology of adjuvant (CaPNs) NPs was analyzed by SEM, showing that most of them were circular in shape with a smooth surface (**Figure 2D**).

### Humoral Immune Responses Induced by Vaccination

**Figure 3:**
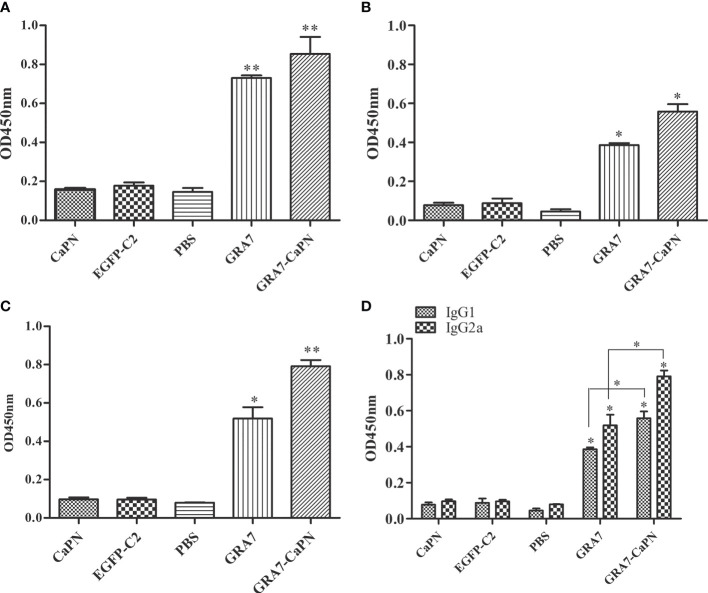
Specific immunoglobulin G (IgG) and IgG isotype analysis. **(A)** Total IgG. **(B)** IgG1. **(C)** IgG2a. **(D)** Levels of IgG1 and IgG2a. Results are represented as the means of OD 450 nm ± SD. *P < 0.05, ** *P <* 0.01. The labels “GRA7” and “GRA7-CaPN” in **Figures 3**–**7** were the abbreviations of “GRA7- pEGFP-C2” and “GRA7- pEGFP-C2-CaPNs”.

To determine the *T. gondii*-specific antibody response, sera from all vaccinated mice were collected, and the total IgG and IgG subclasses (IgG1 and IgG2a) were analyzed by ELISA. High levels of IgG were observed in the serum of the vaccine-immunized groups (GRA7-pEGFP-C2+nano-adjuvant, GRA7-pEGFP-C2) (*P* < 0.01); however, no significant difference was observed between these two vaccine groups (*P* > 0.05) (**Figure 3A**). IgG1 in the immunization groups was also significantly elevated compared to that in the control groups (**Figure 3B**) (*P* < 0.05 for both vaccine groups). The GRA7-pEGFP-C2+nanoadjuvant (CaPN) group exhibited a higher level of IgG2a (**Figure 3C**) (*P* < 0.01), and higher levels of IgG2a were also observed in GRA7-pEGFP-C2-immunized mice (*P* < 0.05) than that in the control groups. Meanwhile, the levels of IgG2a were significantly higher than IgG1 in the vaccine groups (*P* < 0.05, compared to control groups), and the ratios of IgG2a/IgG1 were higher in mice immunized with GRA7-pEGFP-C2+nano-adjuvant (CaPNs) compared to those immunized with GRA7-pEGFP-C2 alone (*P* < 0.05) (**Figure 3D**). Taken together, these results showed that a Th1-type immune response was elicited in response to nanoadjuvant vaccine immunization.

### Cellular Immune Responses

**Figure 4:**
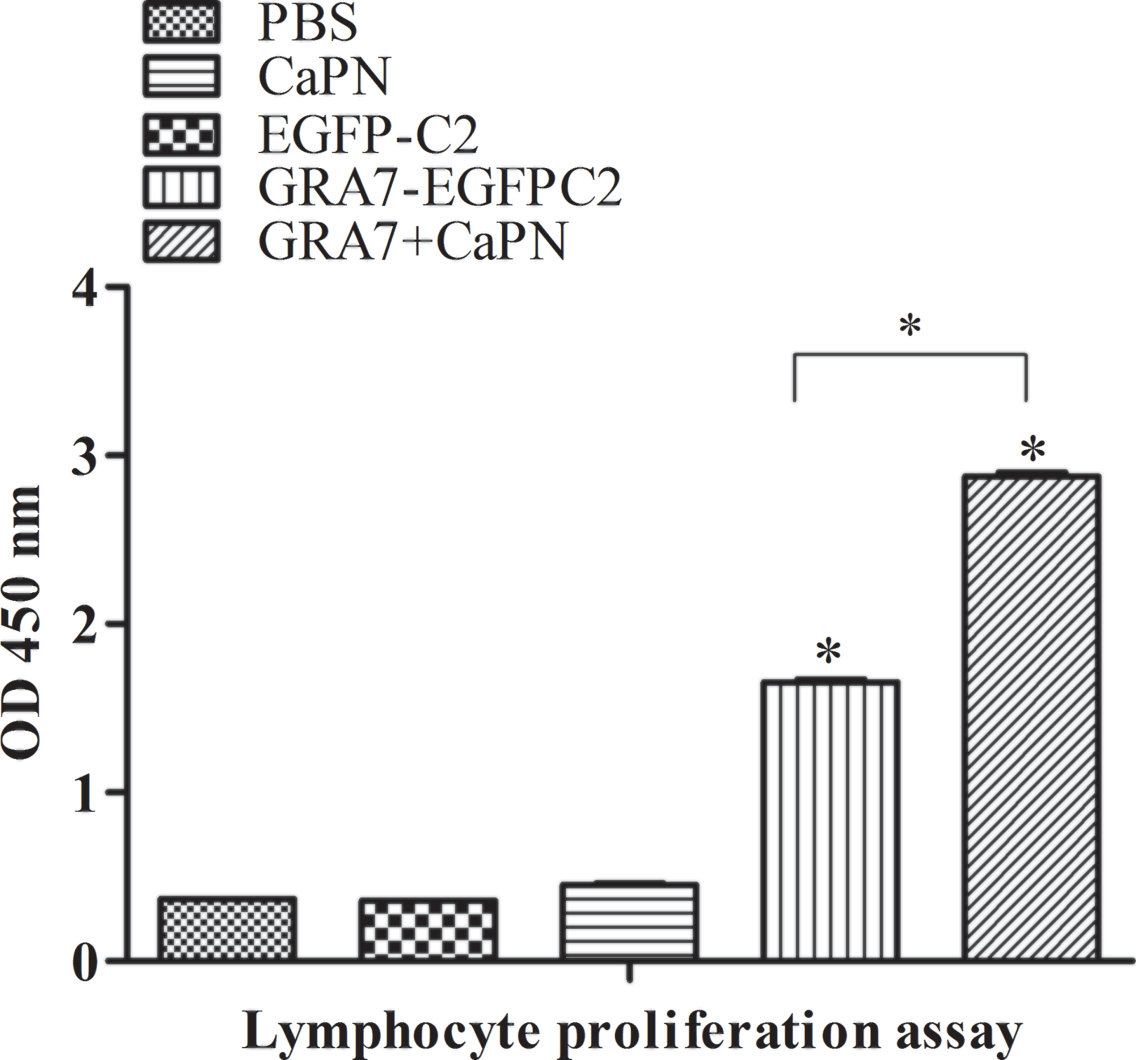
Splenocyte proliferation response in BALB/c mice. Splenocytes from immunized mice and non-immunized mice were collected 63 days after immunization, and the proliferation response was analyzed by Cell Counting Kit (CCK)-8 assay. The data are shown as the means ± *SD* of three independent experiments. *P < 0.05.

Splenocytes were collected from immunized and control groups (5 weeks after the last immunization) to analyze their proliferation, and the cells were treated with TLA and ConA. As shown in **Figure 4**, a significantly higher lymphocyte proliferation SI was obtained in the GRA7-pEGFP-C2+nano-adjuvant (CaPNs) and GRA7-pEGFP-C2 groups compared to the control groups (*P* < 0.05). In addition, the GRA7-pEGFP-C2+nano-adjuvant (CaPNs) group induced an almost 2-fold higher level of lymphocyte proliferation than the GRA7-pEGFP-C2 immunization group (*P* < 0.05).

### Cytokine Responses

**Figure 5:**
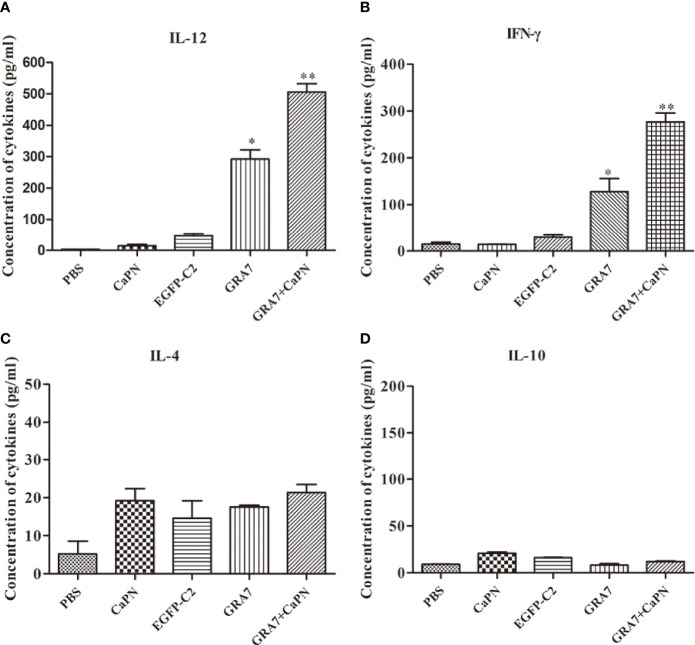
Levels of cytokines produced by splenocyte culture supernatants. **(A)** Production of interleukin (IL)-12 collected from splenocyte supernatants after culture for 96 h. **(B)** Detection of interferon (IFN)-γ collected from splenocyte supernatants. **(C)** Production of IL-4 after culture for 24 h. **(D)** Production of IL-10 after culture for 72 h. *P < 0.05, ** *P <* 0.01.

To further explore T-cell responses to vaccination, splenocytes were collected 63 days after immunization, and supernatants were harvested to evaluate the expression of cytokines, including IL-12, IFN-γ, IL-4, and IL-10 (**Figure 5**). Compared with the control groups, the IL-12 level of mice vaccinated with GRA7-pEGFP-C2+nano-adjuvant (CaPNs) was statistically higher (*P* < 0.01) (**Figure 5A**). Moreover, the production of IFN-γ was significantly higher in the GRA7-pEGFP-C2+nano-adjuvant (CaPNs) group than that in the control groups (*P* < 0.01), and GRA7-pEGFP-C2-treated mice displayed higher levels of IFN-γ (*P* < 0.05) (**Figure 5B**). In contrast, IL-4 and IL-10 levels showed no statistically significant differences in any of the groups compared to the control groups (*P* > 0.05) (**Figures 5C, D**). These results confirmed that immunization with GRA7-pEGFP-C2+nano-adjuvant (CaPNs) or GRA7-pEGFP-C2 promoted a Th1-type immune response.

### Protection From the Recombinant DNA Vaccine

**Figure 6:**
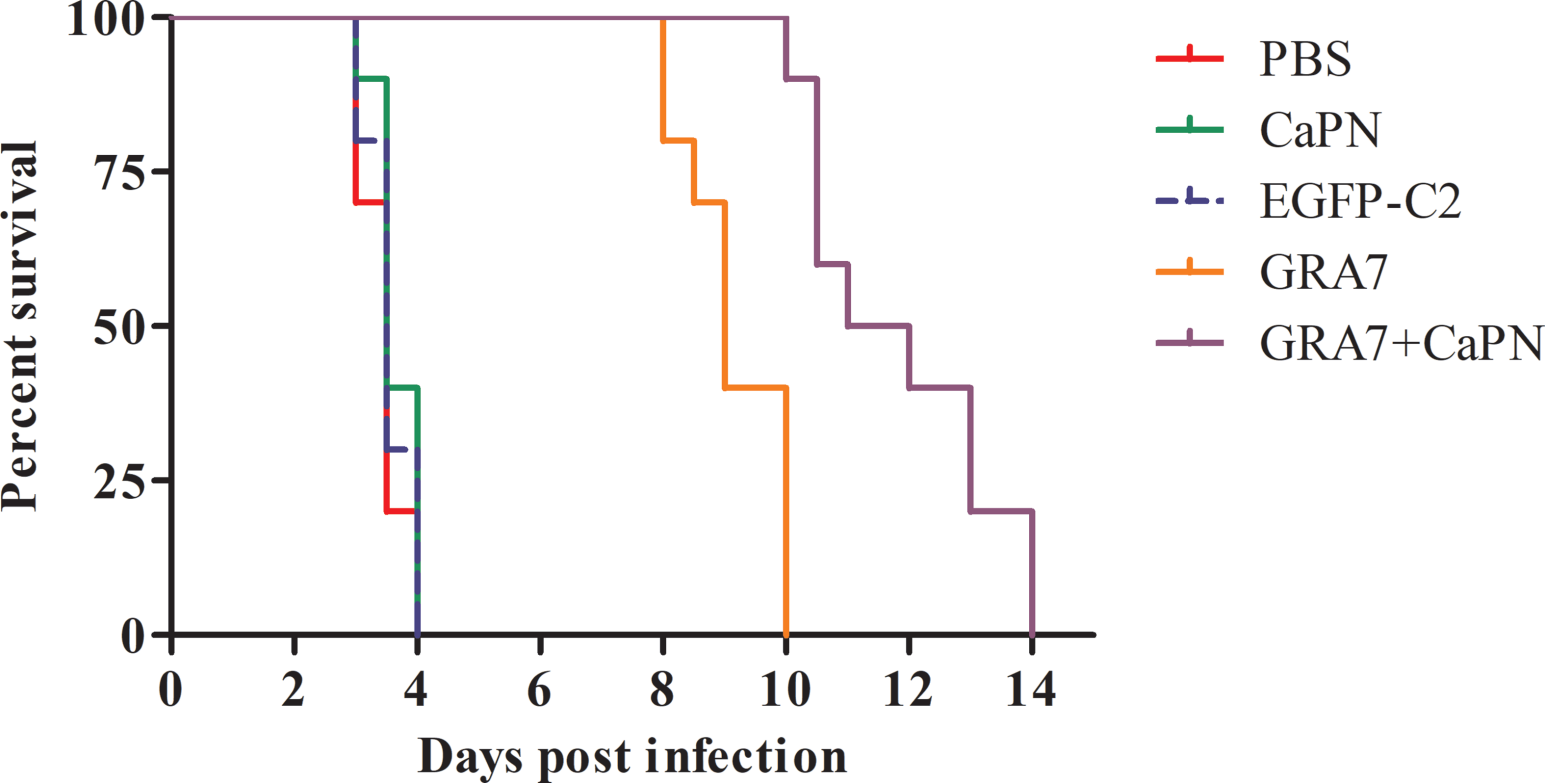
Survival time of BALB/c mice after challenge with *Toxoplasma gondii* tachyzoites. Each group contained 10 mice, and survival was significantly higher in the GRA7-pEGFP-C2+nano-adjuvant (CaPNs)–immunized mice than that in control mice.

To evaluate protective efficacy, mice from all groups were intraperitoneally challenged with 10^4^ tachyzoites of the *T. gondii* RH strains 5 weeks after the final vaccination, and the survival time was recorded. As shown in **Figure 6**, mice in the control groups all died within 4 days (*P* > 0.05), while the survival times of mice immunized with GRA7-pEGFP-C2+nano-adjuvant (CaPNs) (extending survival time to the 14th day) were significantly longer by comparison (*P* < 0.05); however, no significant difference was observed between the GRA7-pEGFP-C2+nano-adjuvant (CaPNs) and GRA7-pEGFP-C2 groups.

### Parasite Burden

**Figure 7:**
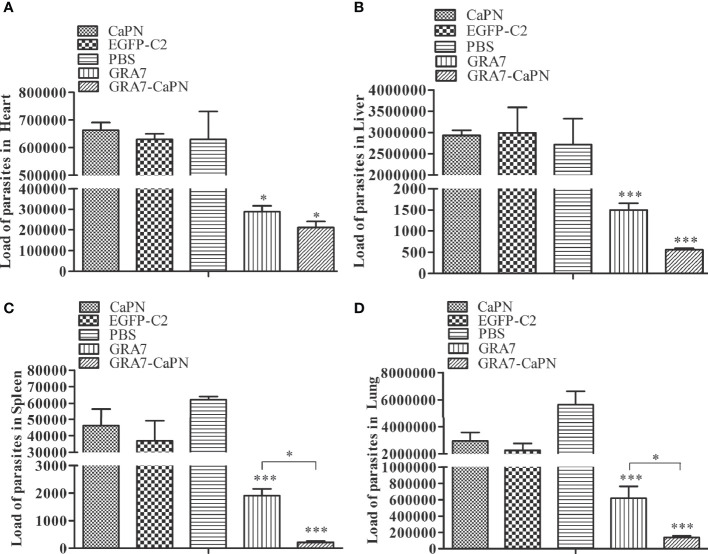
Parasite burden in heart, liver, spleen, and lung tissues in the vaccine and control groups. **(A)** Parasite burden in the heart. **(B)** Parasite burden in the liver. **(C)** Parasite burden in the spleen. **(D)** Parasite burden in the lung. The data are shown as means ± *SD* for three experiments. *P < 0.05, *** *P <* 0.001.

Three mice from each group were randomly selected after death to determine the parasite burden in tissues of *T. gondii*-infected mice in the heart, liver, spleen, and lung. SYBR-green real-time PCR was used to quantify parasite loads. As shown in **Figure 7**, all tissues examined were *T. gondii* infection positive, but the vaccine immunization groups exhibited lower parasite loads than the control groups. Mice immunized with GRA7-pEGFP-C2+nano-adjuvant (CaPNs) or GRA7-pEGFP-C2 exhibited significantly reduced parasite loads in the liver, spleen, and lung (*P* < 0.001) (**Figures 7B–D**), and the average parasite loads in the heart (GRA7-pEGFP-C2+nano-adjuvant group) were reduced by 3.15-fold (*P* < 0.05) compared with the control groups (**Figure 7A**). GRA7-pEGFP-C2+nano-adjuvant (CaPNs)-immunized mice showed decreased parasite loads compared to the GRA7 pEGFP-C2-immunized group in the spleen and lung (*P* < 0.05), and no significant differences were found in the control groups (*P* > 0.05).

## Discussion

DNA vaccines have been considered an effective approach for inducing protection against challenge infections, with the ability to simultaneously elicit both humoral and cellular immune responses (Matowicka-Karna et al., 2009; Wang et al., 2011). DNA vaccines have many advantages, such as the ease of constructing recombinant plasmids and native protein structures, ensuring appropriate processing and immune presentation (Li and Petrovsky, 2016). Although DNA vaccines can trigger an immune response, these vaccines have often been poorly immunogenic due to various factors, such as poor distribution of plasmids (Verma and Khanna, 2013; Zhang et al., 2013), inefficient expression, or rapid degradation by lysosomes and DNase. To improve DNA vaccine immunogenicity, novel adjuvants have been explored (Petrovsky and Aguilar, 2004). NPs are promising adjuvants that can deliver antigens to certain cells (Van Riet et al., 2014) and trigger an immune response to vaccine antigens (De Koker et al., 2011; Kasturi et al., 2011). Many studies have shown that NPs can modulate cellular and humoral immune responses, such as poly (gamma-glutamic acid) NPs (Okamoto et al., 2009), novel core–shell nanospheres and microspheres (Caputo et al., 2009), new cationic NPs (Debin et al., 2002), and CaPNs (Ahmadpour et al., 2017). A specific anti-*T. gondii* antibody response contributes to killing engulfed parasites (Xu et al., 2014), and several adjuvants have been used to enhance immune responses against *T. gondii* infection. CaPNs are known to be biocompatible and non-cytotoxic and can be efficient adjuvant materials for antigen delivery systems (Zhang et al., 2012). The Ca^2+^ and PO4^3-^ ions can participate in the normal metabolism of organisms (Wang et al., 2020). CaPNs loaded with different types of nucleotide chains have been widely used as nano-platforms for gene, drug, and vaccine delivery systems (Zhou et al., 2017; HeBe et al., 2019). Calcium is highly effective in condensing DNA because a small hydrodynamic radius prompts a high charge-to-surface area (Kulkarni et al., 2006). Calcium phosphate (CAP) has a proper adjuvant potential in enhancing immune responses against different infectious illnesses (Lin et al., 2017). Previous study has shown that CaPNs were a potent antigen delivery system to immunize brucellosis compared with aluminum hydroxide (AH) and chitosan (CS) NPs (Abkar et al., 2019).

The size and morphology of CaPs greatly affect their transfection efficiency (Neumann et al., 2009), their ability to bind to specific cell membrane receptors, their trafficking inside the cells, and their intracellular flow (Jiang et al., 2008; Liu et al., 2011). All particles used in vaccine formulations typically have comparable size (Pedraza et al., 2008), and the mechanism by which NPs (20–200 nm in diameter) are taken up is typically endocytosis; larger particles (0.5–5 μm) are taken up by micropinocytosis, while particles above 0.5 μm are thought to be taken up by phagocytosis (Xiang et al., 2006). The average diameter of CaPNs in our study was 47.28 nm (**Figure 2A**), which was suitable for delivery of DNA into cells through endocytosis and thereby enhanced the immune response.

In the present study, mice injected with the GRA7-pEGFP-C2+nano-adjuvant (CaPNs) vaccine developed a significant level of *T. gondii*–specific total IgG and a higher IgG2a-to-IgG1 ratio. Elevated IgG2a is an indicator of a Th1-based immune response, while IgG1 indicates the development of a Th2 immune response. These results were confirmed by the results of the cytokine assay conducted on spleen cell culture supernatants, in which the expression levels of IL-12 and IFN-γ (Th1-type cytokine) in mice immunized with GRA7-pEGFP-C2+nano-adjuvant (CaPNs) (*P* < 0.01) or GRA7-pEGFP-C2 (*P* < 0.05) were significantly higher than those in the control mice (**Figure 5**), while mice in the GRA7-pEGFP-C2+nano-adjuvant injection group produced higher IFN-γ levels than those in the GRA7-pEGFP-C2 immunization group. We suggest that the presence of CaPNs as a nano-adjuvant within pcGRA7 provided an immunogenic antigen and induced a high antibody response. IL-12 leads to the release of IFN-γ and induces the differentiation of Th1 T lymphocyte response to control *T. gondii* infection; disruption of IL-12 expression promoted *T. gondii* growth and dissemination because of diminishing Th1 immune responses (Morgado et al., 2014). The Th2-type cytokines, IL-4 and IL-10, were not induced by immunization with the vaccine (*P* > 0.05). IL-4 is vital for inhibiting severe immunopathology during both the acute and chronic phases of *T. gondii* infection (Denkers and Gazzinelli, 1998), and IL-4 is generally antagonistic to IFN-γ and plays important roles in early *T. gondii* infection (Hunter and Sibley, 2012). Elevated IFN-γ production and low IL-4 levels were also detected in mice injected with the ROP18 multi-epitope DNA vaccine plus the IL-12 plasmid as a genetic adjuvant, and coadministration of pcIL-12 with multi-epitope ROP8 enhanced the levels of IgG antibody and the IgG2a-to-IgG1 ratio (Foroutan et al., 2020). The use of a genetic adjuvant successfully enhanced the protection level. As mice immunized with the ROP13-GRA14-alum nano-adjuvant exhibited significant production of IL-4 and IgG1, the Th2 immune response was developed by immunization with a DNA vaccine coated with alum nano-adjuvant (Pagheh et al., 2021). Mouse priming with GRA1 DNA vaccine-loaded chitosan particles resulted in high anti-GRA1 antibodies and a higher IgG2a/IgG1 ratio (Bivas-Benita et al., 2003).

Specific T-lymphocyte activation (CD4^+^ and CD8^+^ T cells) may play an important role in controlling *T. gondii* infection. CD8^+^ T cells are specialized cytotoxic T lymphocytes that mediate lysis of *T. gondii* through the production of IFN-γ (Dupont et al., 2012); in other words, IFN-γ promotes the acquired cell-mediated immune response by directly acting on CD8^+^ T cells (Grover et al., 2012). In the present study, we found that a significantly higher level of splenocyte proliferation was induced by GRA7-pEGFP-C2+nano-adjuvant (CaPNs) immunization (**Figure 4**), which indicated that an activated cellular immune response was induced in the vaccine immunization group and that increased proliferation of lymphocytes was induced by coating with CaPNs compared to mice immunized with GRA7 alone (*P* < 0.05). A vigorous lymphocyte proliferation effect was observed in mice immunized with pcGRA14+rGRA14-CaPNs compared to mice immunized with GRA14 alone, indicating that enhancement of humoral and cellular immune responses and the protective effects were induced by CaPNs (Pagheh et al., 2019).

No effective vaccine has been shown to completely protect against infection by the *T. gondii* RH strain (Johnson et al., 2004), so the survival rates of immunized mice challenged with a lethal dose (1 × 10^4^) of tachyzoites were analyzed in the present study. The findings indicated that mice immunized with GRA7-pEGFP-C2+nano-adjuvant (CaPNs) or GRA7-pEGFP-C2 vaccine exhibited extended survival time compared to control groups. Control mice all died within 4 days, while those immunized with GRA7-pEGFP-C2+nano-adjuvant (CaPNs) survived for significantly longer, indicating that GRA7 induces partially effective protection in mice against acute *T. gondii* infection and that CaPNs increase protection against *T. gondii* infection, in agreement with the research by Pagheh et al. (2019). In another study, *T. gondii* nucleoside triphosphate hydrolase-II (NTPase-II) coated with lipid NPs showed an increased protective effect against *T. gondii* RH strain (1 × 10^3^) infection, and a significantly prolonged survival time was observed compared to immunization with the NTPase-II vaccine alone (Luo et al., 2017). Various studies have analyzed the presence of *T. gondii* in different tissues of vaccine-injected mice or non-vaccine immunization groups by qualitative PCR to evaluate the protective effect against *T. gondii* infection (Lu et al., 2017; Alizadeh et al., 2019). We investigated the parasite load in the present study. The parasite load in the GRA7-pEGFP-C2 immunization group was significantly decreased compared to that in the control groups and was particularly low in GRA7-pEGFP-C2+nano-adjuvant (CaPNs)-immunized mice (**Figure 7**). GRA7-pEGFP-C2+nano-adjuvant (CaPNs)-immunized mice displayed decreased parasite loads compared to those in the GRA7-pEGFP-C2-immunized group in the spleen and lung (*P* < 0.05).

In conclusion, in the work presented herein, we presented a nano-particulate vaccine, GRA7-pEGFP-C2+nano-adjuvant (CaPNs). *T. gondii* GRA7 coated with CaPNs induced a significant level of *T. gondii*–specific total IgG and a higher IgG2a-to-IgG1 ratio. CaPNs enhanced splenocyte proliferation, elevated IL-12 and IFN-γ production, and decreased IL-4 levels in mice injected with the GRA7-pEGFP-C2+nano-adjuvant (CaPNs) vaccine. GRA7-CaPN-immunized mice exhibited markedly longer survival times and decreased parasite loads compared to mice immunized with GRA7 alone. Taken together, these results indicated that CaPN-based immunization with *T. gondii* GRA7 represents a promising approach for improving vaccination.

## Data Availability Statement

The original contributions presented in the study are included in the article/supplementary material. Further inquiries can be directed to the corresponding author.

## Ethics Statement

The animal study was reviewed and approved by the Animal Ethics Committee of Zhejiang Academy of Agricultural Sciences.

## Author Contributions

H-CS and T-YS conceived and supported the study. H-CS wrote the article. JH and YF performed the experiments. L-LH and XL analyzed the data. All authors contributed to the article and approved the submitted version.

## Funding

This work was supported by the National Natural Science Foundation of China (31802183), Zhejiang Province “Sannongliufang” Science and Technology Cooperation Project (Grant No. 2020SNLF007), and the National Natural Science Foundation of China (32072883).

## Conflict of Interest

The authors declare that the research was conducted in the absence of any commercial or financial relationships that could be construed as a potential conflict of interest.

## Publisher’s Note

All claims expressed in this article are solely those of the authors and do not necessarily represent those of their affiliated organizations, or those of the publisher, the editors and the reviewers. Any product that may be evaluated in this article, or claim that may be made by its manufacturer, is not guaranteed or endorsed by the publisher.
